# Boiling and *Bacillus* Spores

**DOI:** 10.3201/eid1010.040158

**Published:** 2004-10

**Authors:** Eugene W. Rice, Laura J. Rose, Clifford H. Johnson, Laura A. Boczek, Matthew J. Arduino, Donald J. Reasoner

**Affiliations:** * U.S. Environmental Protection Agency, Cincinnati, Ohio, USA;; † U.S. Centers for Disease Control and Prevention, Atlanta, Georgia, USA

**Keywords:** letter, boiling, anthrax spores

**To the Editor:** Public health authorities rely upon "boil water" advisories to alert consumers if a potable water supply is deemed unsuitable for consumption. Holding water at a rolling boil for 1 minute will inactivate waterborne pathogens, including encysted protozoa (*1**–**3*). Spores of *Bacillus anthracis*, the agent that causes anthrax, are one of the microorganisms most refractory to inactivation by the boiling water method. This study was conducted to determine the resistance of spores of *B. anthracis* Sterne and three other strains of *Bacillus* spp. in boiling water.

*B. anthracis* Sterne (Colorado Serum Co., Denver, CO) was grown on soil extract peptone beef extract medium (*4*). Spores were harvested from the agar plates and washed four times by centrifugation with sterile distilled water, treated with 50% (vol/vol) ethanol while being shaken at 100 rpm for 2 h, then washed an additional four times by centrifugation with sterile distilled water. Spores of one of the *B. cereus* strains were obtained from a commercial source (Raven Biological Laboratories, Omaha, NE). Spores were produced in broth cultures for the other *Bacillus* spp. The second *B. cereus* (ATCC 9592) was grown in a generic sporulation medium (*5*), and *B. thuringiensis* var. *israelensis* (ATCC 35646) was grown in Schaefer's medium (*6*). Spores were purified by gradient separation using RenoCal-76 (Bracco Diagnostics, Princeton, NJ) (*6*). Spore preparations were stored in 40% (vol/vol) ethanol at 5°C until used.

Duplicate experiments for each species were conducted in 1-L glass beakers containing 500 mL of municipal drinking water (21±2°C, pH 8.2±0.5, free available chlorine 0.5±0.3 mg/L). The beakers were left uncovered or covered with a watch glass. Steam was allowed to escape from the covered beakers through the mouth of the pouring spout. Water samples were injected with the spore preparations, heated to boiling on a hot plate, and held at boiling temperature for various times. Measuring the boiling times began when the sample reached a rolling boil. A thermocouple thermometer (Cole-Parmer, Vernon Hills, IL) directly above the liquid-air interface determined the air temperature above the boiling water after 5 min of exposure. At the conclusion of the various boiling times, the samples were removed from the heat source and allowed to cool at room temperature before analysis. These samples contained <0.2 mg/L of free available chlorine. Decimal dilutions of the water samples were analyzed in triplicate by the membrane filter procedure with nutrient agar (*7*).

Spores of all strains of the *Bacillus* spp. analyzed in this study were inactivated after boiling for 3–5 min in a covered vessel (Table). Spores still survived after 5 min of boiling in an open vessel for all of the *Bacillus* spp. Temperatures immediately above the surface of the boiling water in the covered vessels averaged 98.9°C, while the temperature immediately above the water level in the uncovered vessels averaged 77.3°C.

**Table:** Inactivation of *Bacillus* spp. by boiling in tap water

Organism	Initial log_10_ CFU/mL		Boiling times^a^ log_10_ CFU/mL					
			1 Min		3 Min		5 Min	
	Covered	Uncovered	Covered	Uncovered	Covered	Uncovered	Covered	Uncovered
*B. anthracis* Sterne	4.95	4.92	0.11	ND^b^	<0^c^	2.13	<0^c^	2.01
*B. cereus* (commercial)	4.62	4.59	0.81	1.94	<0^c^	1.50	<0^c^	1.46
*B. cereus*, ATCC 9592	4.54	4.76	<0^c^	0.78	<0^c^	0.60	<0^c^	0.48
*B. thuringiensis* ATCC 35646	4.63	4.46	<0^c^	1.76	<0^c^	1.58	<0^c^	1.47

^a^Values are means of duplicate experiments <0.25 log units. ^b^ND, not determined. ^c^<0, a number reading below the detection level.

In a comprehensive literature review citing published reports dating back to 1882, Murray (*8*) noted that boiling times reported to destroy *B. anthracis* spores varied over a range of 1 to 12 min. In his own study of 17 strains of *B. anthracis*, Murray (*8*) found that boiling times of 5 to 10 min were required to achieve inactivation. Stein and Rogers (*9*) reported that vigorous boiling for 3 to 5 min destroyed spores from 43 strains of *B. anthracis*.

In our study, boiling water in a covered vessel for 3 to 5 min destroyed spores of the *Bacillus* spp. by greater than four orders of magnitude. Boiling for 5 min in an uncovered vessel was not as effective as boiling in a covered vessel and allowed all *Bacillus* spp. spores to survive. On the basis of the initial levels of spores used in this study, holding water at a rolling boil for 1–3 min in an open container would not inactivate the spores. Boiling time refers to the total time the water is held at a rolling boil and should not be confused with the first sign of bubbles from dissolved gases in the water. Since water boils at lower temperatures at higher altitudes (approximately 90°C at 3 km), boiling times must also compensate for decreased atmospheric pressure conditions (*1**,**2*).
